# Gallic acid, a common dietary phenolic protects against high fat diet induced DNA damage

**DOI:** 10.1007/s00394-018-1782-2

**Published:** 2018-07-23

**Authors:** Tahereh Setayesh, Armen Nersesyan, Miroslav Mišík, Rahil Noorizadeh, Elisabeth Haslinger, Tahereh Javaheri, Elisabeth Lang, Michael Grusch, Wolfgang Huber, Alexander Haslberger, Siegfried Knasmüller

**Affiliations:** 1 Institute of Cancer Research, Department of Medicine I, Medical University of Vienna, Vienna, Austria; 2 Ludwig Boltzmann Institute for Cancer Research, Vienna, Austria; 3 Institute of Animal Breeding and Genetics, University of Veterinary Medicine Vienna, Vienna, Austria; 4 Department of Nutritional Sciences, University of Vienna, Vienna, Austria

**Keywords:** High fat diet, Obesity, DNA damage, Gallic acid, Inflammation

## Abstract

**Purpose:**

Aim of the study was to find out if gallic acid (GA), a common phenolic in plant foods, prevents obesity induced DNA damage which plays a key role in the induction of overweight associated cancer.

**Methods:**

Male and female C57BL6/J mice were fed with a low fat or a high fat diet (HFD). The HFD group received different doses GA (0, 2.6–20 mg/kg b.w./day) in the drinking water for 1 week. Subsequently, alterations of the genetic stability in blood and inner organs were monitored in single cell gel electrophoresis assays. To elucidate the underlying molecular mechanisms: oxidized DNA bases, alterations of the redox status, lipid and glucose metabolism, cytokine levels and hepatic NF-κB activity were monitored.

**Results:**

HFD fed animals had higher body weights; increased DNA damage and oxidation of DNA bases damage were detected in colon, liver and brain but not in blood and white adipose tissue. Furthermore, elevated concentrations of insulin, glucose, triglycerides, MCP-1, TNF-α and NF-κB activity were observed in this group. Small amounts of GA, in the range of human consumption, caused DNA protection and reduced oxidation of DNA bases, as well as biochemical and inflammatory parameters.

**Conclusions:**

Obese animals have increased DNA damage due to oxidation of DNA bases. This effect is probably caused by increased levels of glucose and insulin. The effects of GA can be explained by its hypoglycaemic properties and indicate that the consumption of GA-rich foods prevents adverse health effects in obese individuals.

**Electronic supplementary material:**

The online version of this article (10.1007/s00394-018-1782-2) contains supplementary material, which is available to authorized users.

## Introduction

Obesity causes metabolic disorders and cardiovascular diseases and is also associated with higher rates of cancer in multiple organs and possibly with infertility [1, 2]. It is known that DNA damage plays a crucial role in malignant transformation of cells and affects reproductive success [3, 4]. Nevertheless, only few studies have been published which concern the role of genetic instability in regard to adverse health effects caused by excessive body weight [5–8]. Results of intervention trials with humans and animals indicate that weight loss and intake of specific dietary constituents (e.g. vitamin E and epigallocatechin gallate) may reduce obesity-associated DNA instability; however, significant effects were only seen with high doses of the individual compounds which are not contained in the regular human diet [9–11].

The aim of the present study was to investigate the impact of obesity on DNA instability in an animal model which reflects the metabolic changes in overweight humans [12] and to study the protective effects of low doses of gallic acid (GA), a common dietary phenolic which is contained in a number of plant foods and beverages [13]. To elucidate the molecular mechanisms of obesity induced DNA damage and the potential protective effects of GA, we measured also oxidation of DNA bases and a number of biochemical markers which reflect the redox status and inflammation. It is generally assumed that overweight leads to systemic inflammation and the formation of reactive oxygen species (ROS) which are known to cause damage of biologically relevant molecules including DNA [2, 14].

In the present study, we fed a high fat diet (HFD) to C57BL/6J mice under controlled conditions. It is well documented that these animals develop obesity, insulin resistance and hypoglycaemia in a way that resembles the metabolic syndrome in humans [15]. It was postulated that this model reflects the effects of Western diet induced obesity [12] in humans and it was used successfully in earlier investigations concerning the impact of excess body fat on DNA instability [9, 11, 16].

Gallic acid (GA) was used in the present trial, since earlier studies indicate that it is an extremely potent dietary antioxidant which is contained in many plant derived foods (blueberries and strawberries, mangos) and also in spices (sumac) [13, 17]. We found that low doses, which correspond to the daily human intake in Europe, protect healthy individuals as well as diabetic patients against oxidation of DNA bases [13, 18]. Also in experiments with normal weight mice, evidence for the reduction of basal and ROS-induced oxidation of DNA bases was observed [13]. In the present study, the animals were treated with doses, which are similar to the average daily uptake of humans [19–21].

Induction of DNA damage was measured in single cell gel electrophoresis (SCGE) assays which are based on the determination of migration of DNA in an electric field and detect induction of single and double strand breaks and apurinic sites [22]. This method is at present one of the most widely used techniques in genetic toxicology [23]. The oxidation of DNA bases was monitored by use of a modified protocol with repair enzymes [24].

To investigate the impact of obesity and GA consumption on the redox status, several further parameters were monitored including the levels of thiobarbituric acid reactive substances (TBARs) which reflect lipid peroxidation processes, reduced glutathione (GSH) and glutathione peroxidase activity (GPx) [25]. Furthermore, we determined also the concentrations of glucose, insulin and triglycerides, since it was postulated that an increase of these biochemical parameters is causally related to ROS formation [26, 27]. As it is assumed that the adverse health effects of overweight are linked to inflammation [2], we determined also the activity of nuclear factor-κB (NF-κB) in the liver. This transcription factor regulates the expression of genes which play a crucial role in inflammatory responses [28]. It is known that NF-κB is increased in obese individuals and plays an important role in biochemical processes which are influenced by body weight and insulin levels [29]. Additional parameters which were monitored, include tumour necrosis factor-alpha (TNF-α) [2] and monocyte chemoattractant protein-1 (MCP-1) which reflect inflammation and are increased by obesity, type 2 diabetes and other diseases [2].

## Materials and methods

### Animals and feeding scheme

The animal study was approved by the Ethics Committee of the Medical University of Vienna (BMWFW-66.009/0329-WF/V/3b/2014). Male and female C57BL6/J mice (age 4 weeks old; *n* = 40, Harland laboratory, Italy) were housed in groups of five in standard size ventilated cages at the animal facility of the Institute of Cancer Research, Medical University of Vienna. The mice were maintained at 22 ± 2 °C with a 12-h light/dark cycle and had free access to water and food throughout the study.

After acclimatization for one week, the animals were randomly assigned to two groups and fed either with a low fat diet (LFD; Harlan Laboratories inc., teklad TD.120455 low fat diet containing 3.3 kcal/g, 6.2 g animal fat, 50.7 g carbohydrates and 18.6 g protein per 100 g) or a HFD (Harlan Laboratories inc., teklad TD.06414 containing 5.1 kcal/g, 34.3 g animal fat, 27.3 g carbohydrates and 23.5 g protein per 100 g). We used the LFD, as it was suggested as an “ingredient-matched” diet for HFD-studies by the producer [30]. Notably, the slightly higher protein level had only a marginal impact on the overall calorie intake (less than 2%). According to the information from the producer, the amounts of saturated, monounsaturated and polyunsaturated fat in the HFD were 125, 160 and 54 g/kg of the diet, respectively, the n-6:n-3 ratio was 8.8. The composition of the diets is specified in detail in Supplementary Table 1.

After a feeding period of 14 weeks, the HFD group was randomly divided into three subgroups (*n* = 10, five animal per gender). Two groups received different doses of GA (Sigma-Aldrich, CAS No. 149-91-7, purity 99%, Germany) in the drinking water for 1 week; (2.6 or 20 mg/kg b.w./day). The acid was dissolved in drinking water and fresh solutions were prepared every day as the phenolic is stable in room temperature for 24 h [13]. The GA doses (2.6 and 20 mg/kg b.w./day) which were given to the mice correspond to daily consumption of 12.8 and 97.2 mg/person, respectively [31]. Control animals received pure water, no differences of the consumption of pure and GA-supplemented water was observed (see Table 1). The intake of GA supplemented water was determined daily and the amount of GA which was consumed was calculated on the basis of the individual body weights of the animals. To exclude that GA supplemented water was spilled during the consumption, the dryness of the cages was checked routinely and no loss could be detected.

**Table 1:** Impact of HFD, LFD and GA supplementation on alteration of body and organ weights, composition levels and redox parameters of male and female mice (*n* = 5 per group)

Parameters	Groups						
	LFD	HFD	Δ^a^ (%)	HFD + 2.6 GA	Δ^b^ (%)	HFD + 20GA	Δ^c^ (%)
Body and organ weight							
Initial body weights (g)							
♂	18.6 ± 1.9	19.4 ± 1.3	+ 4.3	17.8 ± 1.1	− 8.2	17.4 ± 0.9	− 10.3
♀	15.0 ± 1.0	14.6 ± 0.9	− 2.7	15.8 ± 0.4	+ 8.2	15.4 ± 1.1	+ 5.4
Final body weights (g)							
♂	26.0 ± 4.3	41.8 ± 3.4	+ 60.7^e^	38.8 ± 1.3	− 7.1	43.6 ± 5.9	+ 12.3
♀	23.0 ± 1.6	27.8 ± 1.1	+ 20.8^e^	30.8 ± 5.2	+ 10.7	27.8 ± 0.4	0
Body weight gain (g)							
♂	8.4 ± 2.5	22.4 ± 4.1	+ 166.6^e^	21.0 ± 2.3	− 6.2	26.2 ± 5.4	+ 24.7
♀	8.0 ± 1.9	13.2 ± 1.5	+ 60.0^e^	15 ± 5.2	+ 17.1	12.4 ± 1.3	− 3.2
Liver weight^d^							
♂	1.3 ± 0.2	2.1 ± 0.6	+ 61.5^e^	1.4 ± 0.1	− 33.3^f^	1.5 ± 0.3	− 28.2
♀	0.9 ± 0.1	1.1 ± 0.2	+ 22.2^e^	1.1 ± 0.1	0	1.1 ± 0.1	0
WAT weight^d^							
♂	0.6 ± 0.4	3.2 ± 0.2	+ 433.3^e^	2.2 ± 0.8	− 31.2^f^	2.5 ± 0.9	− 21.9
♀	0.5 ± 0.4	2.6 ± 0.4	+ 420.0^e^	1.7 ± 0.4	− 34.6^f^	1.6 ± 0.6	− 38.5^f^
Consumption levels							
Water consumption (ml/day/mouse)							
♂	3.8 ± 0.7	3.7 ± 1.0	− 2.7	3.9 ± 0.9	+ 5.4	3.7 ± 1.1	0
♀	3.2 ± 0.7	3.6 ± 0.8	+ 12.5	3.0 ± 1.1	− 16.7	3.3 ± 1.0	− 8.4
Food intake (g/day/mouse)							
♂	3.5 ± 0.5	2.5 ± 0.5	− 28.6^e^	2.6 ± 0.3	+ 4.0	2.4 ± 0.4	− 7.7
♀	2.8 ± 0.2	2.1 ± 0.3	− 25.0^e^	2.3 ± 0.3	+ 9.5	2.1 ± 0.2	0
Energy intake (Kcal/day/mouse)							
♂	11.7 ± 1.7	12.1 ± 1.0	+ 3.4	13.5 ± 2.6	+ 11.5	12.5 ± 1.4	− 7.5
♀	9.2 ± 0.7	10.7 ± 1.0	+ 16.3	11.5 ± 1.7	+ 7.4	10.7 ± 1.0	0
Redox parameters							
GSH (µmol/g liver)							
♂	7.9 ± 0.9	7.6 ± 0.7	− 3.8	9.2 ± 1.6	+ 21.0	7.8 ± 0.4	+ 2.6
♀	6.6 ± 0.6	6.8 ± 0.4	+ 3.3	7.0 ± 0.6	+ 2.9	6.6 ± 0.7	− 2.9
GPx (Δ µmol NADPH /mg protein per min)							
♂	1.2 ± 0.1	1.3 ± 0.2	+ 8.3	1.3 ± 0.4	0	1.3 ± 0.0	0
♀	1.4 ± 0.6	1.4 ± 0.1	0	1.5 ± 0.8	+ 7.1	1.4 ± 0.2	0
TBARs (nmol/mg protein)							
♂	0.3 ± 0.07	0.4 ± 0.02	+ 33.3	0.4 ± 0.03	0	0.4 ± 0.03	0
♀	0.6 ± 0.02	0.5 ± 0.04	− 16.7	0.5 ± 0.09	0	0.5 ± 0.05	0

LFD, low fat diet; HFD, high fat diet; HFD + 2.6GA, HFD + 2.6 mg/kg b.w./day; HFD + 20GA, HFD + 20 mg/kg b.w./day; WAT, White adipose tissue

Differences were considered as significant when *P* values were ≤ 0.05 (with Kruskal–Wallis test, Dunn’s Multiple Comparison Test), Numbers indicate means ± SD

Δ-values indicate difference in %

Δ^a^-differences between LFD and HFD

Δ^b^ differences between HFD and HFD + 2 GA

Δ^c^ differences between HFD and HFD + 20 GA

^d^At the end of the experiment

^e^Differences between LFD and HFD groups

^f^Differences between HFD- control (no GA) and GA-supplementation groups

During the initial feeding period (14-weeks), five animals were kept in one cage, during the short GA intervention, two to three animals were kept per cage. Body weights were recorded once per week; food intake and water consumption were measured daily in all groups (see Table 1). The animals were sacrificed by cervical dislocation after a feeding period of 15 weeks; subsequently blood and tissues (i.e. liver, colon, brain and white adipose tissue (WAT)) were collected. WAT-weights were determined as described by Parlee et al. [32]. Briefly, epididymal WAT tissue from the left fat pad, mesenteric tissue around the intestines and retroperitoneal WAT from the back of the kidneys were collected. Subsequently, non-adipose-associated material from the depot including glands and lymph nodes was removed and total wet WAT was weighed immediately after removal with an analytical balance. Part of the samples was frozen immediately after collection in liquid nitrogen for further biochemical analyses.

### Single cell gel electrophoresis assays (SCGE)/comet assay

The experiments were conducted according to the guidelines for SCGE experiments (for details see [22, 33]). Blood and inner organs were analysed according to the method of Sasaki et al. [34]. Blood cells (10 µl whole blood with heparin) were embedded in 90 µl of 0.75% (w/v) low melting point agarose (LMA, 0.5, Gibco, Paisley, UK). The mixtures were transferred to microscope slides pre-coated with 1.5% (w/v) normal melting point agarose (NMA, 1.0, Gibco, Paisley, UK) and topped with a coverslip. Brain, liver and WAT were minced in 4.0 ml chilled homogenization buffer (pH 7.5) and homogenized at 400 rpm on ice with a Potter Elvehjem-type homogenizer (B. Braun, Melsungen, Germany). Subsequently, the homogenates were centrifuged (800×*g*, 10 min, 4 °C). Colon cells were isolated from the mucosa by scraped, kept on ice in 0.5 ml homogenisation buffer and were processed immediately for SCGE analyses [13, 34].

Nuclei from different organs were analysed under standard conditions (*i.e*. with alkaline electrophoresis buffer pH > 13). The slides were placed on ice to solidify the agarose, then the coverslips were removed. Next, the slides were immersed in lysis solution (2.5 M NaCl, 100 mM EDTA and 10 mM Tris, pH 10.0–10.5) containing freshly added 1% Triton X-100 and 10% dimethyl sulfoxide (DMSO) at 4 °C overnight.

In experiments with DNA lesion-specific enzymes, nuclei from different organs were treated after lysis with FPG (10U, Sigma-Aldrich, Germany) or with ENDO III (10U, Sigma-Aldrich, Germany). Both enzymes were calibrated before the main experiment. The slides were washed twice in enzyme buffer (pH 8.0) for 8 min. After calibration of the enzymes, 50 µl of FPG or ENDO III solutions (10–5 µg/ml for liver and 10–7 µg/ml for blood, colon, brain and WAT) or enzyme buffer alone were added to the nuclei. The incubation time for FPG was 30 min and for ENDO III 45 min, respectively. Subsequently, the slides were incubated in fresh alkaline solution (300 mM NaOH and 1.0 mM EDTA, pH > 13) for DNA unwinding for 30 min, then electrophoresis was performed in the same buffer. The electrophoresis conditions were 300 mA and 25 V (0.7 V/cm) for 30 min. All steps were carried out under indirect light and on ice. Following electrophoresis, slides were neutralized in 400 mM Tris buffer (pH 7.5), washed in distilled water and dried overnight. The gels were stained with propidium iodide (Sigma-Aldrich, Germany, 20 µg/ml) air dried and coded for blind analysis. From each tissue and also from the blood, three gels were prepared per animal and from each 50 cells were evaluated. Comet formation was analysed under a fluorescence microscope (Nikon EFD-3, Tokyo, Japan) with 40-fold magnification. DNA migration was determined with a computer-aided image analysis system (Comet Assay IV, Perceptive Instruments, Bury St Edmunds, UK). The percentage of DNA in tail was monitored as a parameter of comet formation.

### Measurement of glutathione (GSH), glutathione peroxidase (GPx) and thiobarbituric acid reactive substances (TBARs)

The levels of hepatic GSH (reduced form) and the activity of GPx were measured spectrophotometrically with DTNB and cumene hydroperoxide as described previously by Huber et al. [35, 36]. TBARs were quantified with trichloroacetic acid and thiobarbituric acid [37]. The protein concentration in each homogenate was estimated by Bradford/Biorad [38]. All samples were measured in duplicate.

### NF-κB activation

For the assessment of nuclear translocation of NF-*κ*B (activation of NF-*κ*B), a commercially available kit (FIVEphoton Biochemical, San Diego, CA, USA) was used to determine the protein in cytosolic and nuclear fractions [39, 40]. The measurements were performed with liver homogenates according to the manufacturer’s protocol. Briefly, following homogenization, cytoplasmic and nuclear protein fractions were isolated. Subsequently, the levels of NF-κB were determined in these fractions by Western blot analysis. Cytoplasmic and nuclear fractions of each sample were diluted (1:100) in Laemmli sample buffer (60 mM Tris–HCl, pH 6.8, 2% sodium dodecyl sulfate (SDS), 1.4% β-mercaptoethanol, 0.005% bromophenol blue and 12% glycerol) and boiled for 5 min. Subsequently, the proteins were size-separated by 10% sodium dodecyl sulfate–polyacrylamide gel electrophoresis (SDS–PAGE, 50 V for 10 min, followed by 120 V for 120 min) and subsequently electro-blotted onto PVDF membranes (Amersham Hyband, GE Healthcare, Germany) using a Bio-Rad (Hercules, CA, USA) tank blotting system. The transfer of proteins was performed at 300 mA for 90 min. The blots were rinsed in PBST (100 mM phosphate buffer, pH 7.5, containing 150 mM NaCl and 0.05% Tween-10) and blocked with 5% non-fat dry milk in PBST for 60 min. Next, the blots were incubated with primary antibody against NF-κB (p65) (1:400) at 4 °C overnight. After washing with PBST, the blots were incubated with goat anti-rabbit IgG–HRP (1:3000) for 2 h and rinsed with PBST. Beta-actin (1:5000, Sigma-Aldrich, Germany) was used as a control. Luminescence was developed using Bio-Rad Clarity Western ECL substrate. Protein bands were recorded using X-ray film or a Bio-Rad ChemiDoc system (Hercules, CA, USA); band intensities were determined with ImageJ software. NF-κB activities were expressed as the relative ratios of nuclear to cytoplasmic p65 band intensities.

### Measurement of biochemical and inflammatory parameters

Triglyceride and glucose concentrations in blood were determined with MultiCare strips (Biochemical Systems International, Italy). Plasma insulin was measured with a mouse ultrasensitive insulin ELISA kit (ALPCO, 80-INSMSU-E01, USA). Circulating levels of inflammatory factors such as TNF-α (Abcam, ab100747, England) and MCP-1 (Abcam, ab100721, England) were monitored with ELISA kits according to the manufacturer’s instructions in plasma and in homogenised liver as described by Zhang et al. [41]. The samples were analysed in duplicate.

### Statistical analysis

Terminal body weights and WAT weights were analysed with one-way analysis of variance (ANOVA) followed by a multiple comparison procedure to compare each treatment to HFD controls (Kruskal–Wallis test, Dunn’s Multiple Comparison Test). DNA damage was monitored in the comet experiments with a Comet Assay image analysis system (Comet Assay IV, Perceptive Instruments, UK). The % tail intensities (%DNA in tail) per animal were determined as suggested by Bright et al. [42]; the comets in each replicate were summarised as median %DNA in tail and then the means of the median %DNA in tail for the three replicates were calculated. The extent of DNA migration attributable to FPG and ENDO III sensitive sites was calculated by subtraction of the corresponding enzyme buffer values which were determined in all experiments. Statistical significance of the comet data was analysed by the non-parametric Mann–Whitney *U* test.

Differences in inflammatory and biochemical markers between groups were evaluated using multifactor ANOVA and multiple-range test (Bonferroni’s method). For all comparisons, results with *p* values < 0.05 were considered significant. Statistical analyses were performed using Graphpad Prism 5.0 (Graphpad Software, San Diego, CA).

## Results

Table 1 summarizes the impact of the feeding schemes on overall body, liver and WAT weights and on food and energy intake in the different groups. It can be seen that the body weights of female and male animals increased after consumption of the HFD; this effect was more pronounced in males. Animals of both sexes consumed higher amounts of the LFD as the corresponding HFD groups and as a consequence no differences were seen in regard to the overall energy intake. Notably, similar results were obtained in earlier feeding studies, *i.e*. the body weights increased after consumption of a HFD diet despite isocaloric consumption of both chows [43]. Also the liver weights of the animals were higher when they received the HFD, furthermore also the WAT weights differed significantly between LFD and HFD animals.

GA supplementation of the drinking water for 7 days did not cause changes of the overall body weight, while WAT was reduced in males and females by 32 and 35%, respectively, after the consumption of 2.6 mg/kg GA b.w./day; a similar effect was also found in the high dose group (20 mg/kg GA b.w./day).

Last part of Table 1 summarizes the alterations of different redox-parameters in hepatic tissue after HFD feeding (with or without GA supplementation). It can be seen that the feeding scheme had no significant impact on the levels of GPx, GSH and TBARs.

**Fig. 1:**
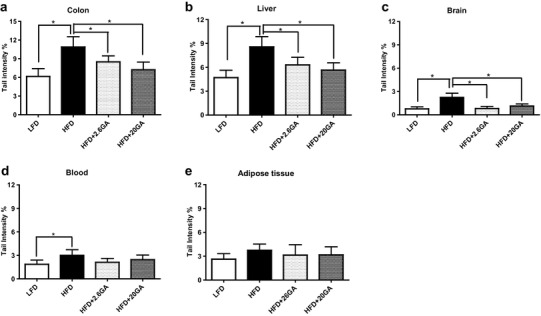
Impact of HFD feeding and GA supplementation on DNA damage in different inner organs. The animals were fed with the diets over a period of 15 weeks; subsequently, the HFD animals received drinking water with and without GA (2.6–20 mg/kg b.w./day). The LFD group received normal drinking water. From each organ, three slides were made and 50 cells were analysed for comet formation per slide. Bars show means of the medians ± SD of results obtained with ten animals (five male and five female) per group. Stars indicate statistical difference (*P* ≤ 0.05, non-parametric Mann–Whitney *U* test)

Figure 1 shows the impact of the HFD on DNA damage in different organs. It can be seen that pronounced effects were observed.

We found increased DNA damage under standard SCGE conditions (which reflect single and double strand breaks and apurinic sites) in colonocytes, hepatocytes, blood and in the brain of the HFD animals. In WAT only a moderate increase of DNA migration was observed in the HFD animals, but this effect did not reach significance.

It is notable that a higher extent of DNA migration was found in colonocytes as in the other organs of lean and obese mice; this observation is in agreement with earlier findings (see for example [11]).

Supplementation of the drinking water with GA led to a decrease of HFD induced DNA damage in liver, brain and colon. These effects were in the latter two organs clearly dose-dependent. The most pronounced protection was seen in cells from the brain and from the gastrointestinal tract; *i.e*. the extent of damage reached the baseline levels which were found in the LFD group. No pronounced sex-specific effects were observed in the different organs.

**Fig. 2:**
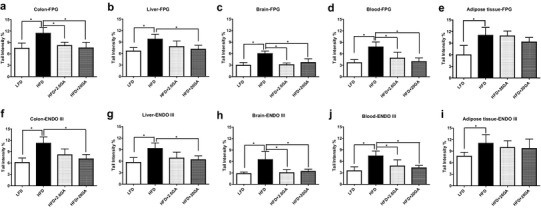
Impact of HFD and GA supplementation of the drinking water on the formation of oxidised purines and pyrimidines in different inner organs of mice. The experiments were conducted as described in materials and methods and in the legend of Fig. 1. Formation of oxidised purines was assessed by treatment of nuclei with formamidopyrimidine DNA glycosylase (FPG); oxidised pyrimidines were determined with endonuclease III (ENDO III). Bars show values obtained with the enzyme after subtraction of results obtained with the respective buffers. From each organ, three slides were made and 50 cells were analysed per slide. Bars show means of the medians ± SD of values which were obtained with ten animals (five male and five female) per group. Stars indicate statistical difference (*P* ≤ 0.05, non-parametric Mann–Whitney *U* test)

Figure 2 summarizes the results of experiments with the lesion specific enzymes (FPG and ENDOIII) which were conducted to assess the extent of oxidation of purines and pyrimidines.

We found in all organs evidence for increased oxidation of DNA bases in the obese (HFD) animals. Supplementation of the drinking water with GA led to a strong reduction of these effects. The extent of protection was similar in blood, colon, liver and slightly more pronounced in the brain. Furthermore, our findings indicate that the effects increase in most tissues with the amount of GA in the drinking water. Again no sex-specific differences were observed.

**Fig. 3:**
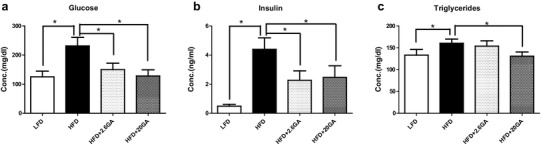
Impact of HFD feeding and GA supplementation on glucose, insulin and triglyceride levels in hepatic tissue and plasma. The animals recieved either a HFD or with a LFD. The animal received drinking water with and without GA (2.6 and 20 mg/kg b.w./day). Bars indicate means ± SD. Stars indicate statistically significance (*P* ≤ 0.05, Bonferroni’s method)

Figure 3 shows the findings of measurements which concerned the glucose and fat metabolism; it can be seen that the levels of glucose, insulin and triglycerides in plasma were increased in the obese HFD animals. GA supplementation led to a pronounced reduction of these effects in both sexes.

**Fig. 4:**
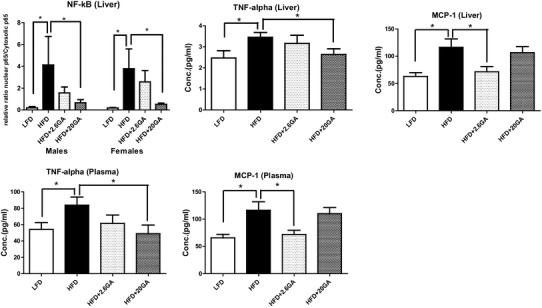
Impact of HFD and GA supplementation on activity of NF-kB and on TNF-α and MCP-1 levels. **a** Represents the relative ratio of nuclear to cytoplasmic in the liver (Western blot image see Supplementary Fig. 1). **b**–**e** Show the levels of TNF-α and MCP-1 in hepatic tissue and plasma. The animals were fed with HFD or LFD and received drinking water with and without GA (2.6 and 20 mg/kg b.w./day). Bars indicate means ± SD. Stars indicate statistical difference (*P* ≤ 0.05, Bonferroni’s method)

HFD-induced obesity led also to the activation of NF-kB in the liver (Fig. 4a, Supplementary Fig. 1s.). The level of the transcription factor was higher in males compared to females of the LFD (control) group (relative ratio in males 0.312 and in females 0.202). This observation is in agreement with findings of earlier study [44]. Supplementation of the drinking water with GA caused clear reduction of the nuclear concentrations of the transcription factor; after administration of the higher dose (20 mg/kg b.w.), the levels were similar to those found in the LFD group. Figure 4b–e summarize alterations of inflammatory parameters which were caused by HFD feeding with and without GA. It is evident that consumption of fat caused a clear increase of TNF-α and MCP-1 levels in plasma and liver. After administration of the phenolic, the concentrations of both markers decreased; the MCP-1 effects were not dose-dependent while the plasma levels of TNF-α decreased in a dose-dependent manner.

## Discussion

Taken together, the results of the present study indicate that obesity leads to DNA damage in colon, liver, blood and brain. Furthermore, no differences were seen between lean and fat animals in WAT. The effects of the HFD diet were paralleled by increased formation of oxidised DNA bases and by changes of the glucose and fat metabolism, an increase of inflammatory markers and alterations of the activity of NF-kB in the liver.

The detection of increased DNA damage in the HFD groups is in agreement with results of previous experiments which focused on liver and colon [9, 11, 45–47], while no effects were detected in the blood [47]. Also in γ-H2AX experiments (which detect DSBs), increased damage was observed in colon and kidney [10] of obese Zucker rats. Furthermore, our data show that excess body weight leads also to pronounced damage in the brain; this observation is in agreement with findings of Langie et al. [48], who measured DNA migration in different parts of the central nervous system of obese rats.

We found evidence for increased oxidative damage of purines and pyrimidines by use of repair enzymes (FPG and ENDOIII) in all organs except in WAT. Notably, measurements of the 8-oxo-2′-deoxyguanosine (8-oxo-dG) levels in brain, urine and liver of obese animals yielded conflicting findings [10, 48]. The group of Stopper et al. [10] found higher level of oxidised DNA and RNA (8-oxoGuo and 8-oxoGua) in the urine of obese HFD rats in comparison to lean animals, while Langie et al. [48] could not detect significant differences in brain regions of obese HFD mice in 8-oxodG measurements. This differences may be due to the tissue specific effects.

We found higher levels of insulin, glucose and triglycerides in the obese animals. Furthermore, HDF feeding caused higher levels of TNF-α, MCP-1 and activation of NF-κB; similar effects were seen in earlier studies [44, 49]. It can not be excluded that saturated fatty acids contained in lard (which was the main source of fat in the HFD diet) may contribute to the induction of pro-inflammatory markers [50].

We found no evidence for changes of hepatic concentrations of GSH, GPx and TBARs. In this context, it is notable that also an earlier investigation did not detect obesity-related induction of the later marker in the liver while a significant alteration was observed in the plasma and muscle of overweight animals, these differences may be due to organ and/or species specific effects [25].

**Fig. 5:**
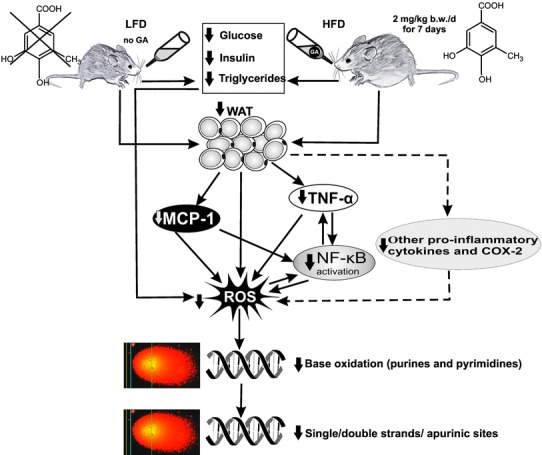
Schematic representation of the molecular mechanisms by which GA leads to DNA protection in HFD-fed mice. Oral administration of the phenolic leads to lower levels of insulin, glucose and triglycerides and in parallel to a reduction of the WAT weights. These processes lead to reduced formation of ROS either directly or indirectly via modulation of the levels of pro-inflammatory chemokines and reduced activation of NF-kB which controls the transcription of genes encoding and for cytokines and COX2. ROS cause oxidative damage of DNA bases and as a consequence induction of single/double strand breaks and apurinic sites which were detected in comet experiments. Solid lines indicate effects which were seen in the present study, dotted lines refer to findings of earlier investigations

Supplementation of the drinking water with small amounts of GA which are similar to the average daily consumption of humans in Germany [19–21] caused pronounced reduction of obesity-induced DNA damage and prevented oxidation of DNA bases. These effects were seen in all organs except in WAT and were paralleled by distinct changes of different biochemical parameters. The results of the present study enable, the development of a plausible hypothesis for the DNA protective properties of GA, in combination with findings from earlier investigations: The induction of DNA damage in different inner organs was paralleled by oxidative damage of purines/pyrimidines, resulting from ROS formation, which is characteristic for excess body weight [51]. Decreased levels of glucose, insulin, triglycerides and also reduction of WAT weights which were found in the present study may lead to reduced generation of ROS and as a consequence to decreased formation of oxidised DNA bases and to protection against obesity-induced induction of single and double strand breaks which was detected in comet experiments under standard condition (Fig. 5).

Notably, a decrease of the glucose levels was observed in a number of earlier studies with GA in HFD fed mice and also in diabetic rats [52–55]. Also decreased concentrations of insulin and triglycerides were detected after administration of the phenolic to rats [55, 56]. Reduction of the fat tissue was found in a trial with fat feeding in mice [56]; furthermore, it is notable that the size of adipocytes was found to decrease as a consequence of administration of GA in HFD fed mice [52, 54, 57].

It is known, that high glucose levels may lead to the formation of ROS via multiple pathways; i.e. via PKC-dependent activation of NADPH-oxidase [58], formation of glycoxidation products [59] and hypoxia [60]. Stopper and co-worker showed that high insulin levels lead to ROS-mediated oxidative damage via activation of the PI3K/Akt pathway [61]. The reduction of the glucose levels by GA can be explained by alterations of different signalling pathways including activation of AMPK/Sirt1/PGC1α [52] and Akt-signalling and increased translocation of glucose transporter-4 may also play a role [54, 62]. The reduction of the adipose tissue is possibly a result of the hyperglycaemic properties of GA and its impact on lipid metabolism [56]. It is known that WAT accumulation in obese subjects leads to stimulation of MCP-1. This causes an increase of the secretion of TNF-α which triggers the activation of NF-*κ*B. This protein controls the transcription of many pro-inflammatory cytokines and of cyclooxygenase*-*2 which play a crucial role in oxidative stress [63].

As mentioned in the introduction, human and animal studies show that increased body weight is associated with induction of DNA damage in multiple organs [6–8, 64] and it is also well documented that damage of the genetic material plays a crucial role in the malignant transformation of cells [3]. In conclusion, the findings of the present study show that obesity leads in multiple inner organs to DNA damage which can be prevented by low amounts of GA which occurs in many plant-derived foods and beverages. This observation is relevant in regard to the development of dietary strategies aimed at preventing the adverse health effects of obesity which has become a world-wide problem.

## Electronic supplementary material

Below is the link to the electronic supplementary material.

Supplementary material 1 (DOC 276 KB)
