# Supplementary material for: Gallic acid, a common dietary phenolic protects against high fat diet induced DNA damage
Source: Eur J Nutr. 2018 Jul 23;58(6):2315–26. doi: 10.1007/s00394-018-1782-2 (PMC6689278; doi:10.1007/s00394-018-1782-2)
Supplement: Supplementary file 1 — Supplementary material 1 (DOC 276 KB) [file 394_2018_1782_MOESM1_ESM.doc]

# Gallic acid, a common dietary phenolic protects against high fat diet induced DNA damage

Tahereh Setayesh^1^, Armen Nersesyan^1^, Miroslav Mišík^1^, Rahil Noorizadeh^1,3^, Elisabeth Haslinger^1^, Tahereh Javaheri^2,3^, Elisabeth Lang^1^, Michael Grusch^1^, Wolfgang Huber^1^, Alexander Haslberger^4^, Siegfried Knasmüller^1^*

1. Institute of Cancer Research, Department of Medicine I, Medical University of Vienna, Vienna, Austria
2. Ludwig Boltzmann Institute for Cancer Research, Vienna, Austria.
3. Institute of Animal Breeding and Genetics, University of Veterinary Medicine Vienna, Vienna, Austria.
4. Department of Nutritional Sciences, University of Vienna, Vienna, Austria

* **Corresponding author**: Siegfried Knasmüller, Institute of Cancer Research, Department of Internal Medicine I, Medical University of Vienna, Vienna, Austria, Tel: +43 1 4016057562; Fax: +43 1 40160957500; E-mail: [siegfried.knasmueller@meduniwien.ac.at](mailto:siegfried.knasmueller@meduniwien.ac.at)

**Acknowledgements** The work was funded by the Austrian Science Funds (Fonds zur Förderung der wissenschaftlichen Forschung (FWF); AP2658721).

**Supplementary Fig. 1** Impact of HFD and GA consumption on NF-kB.


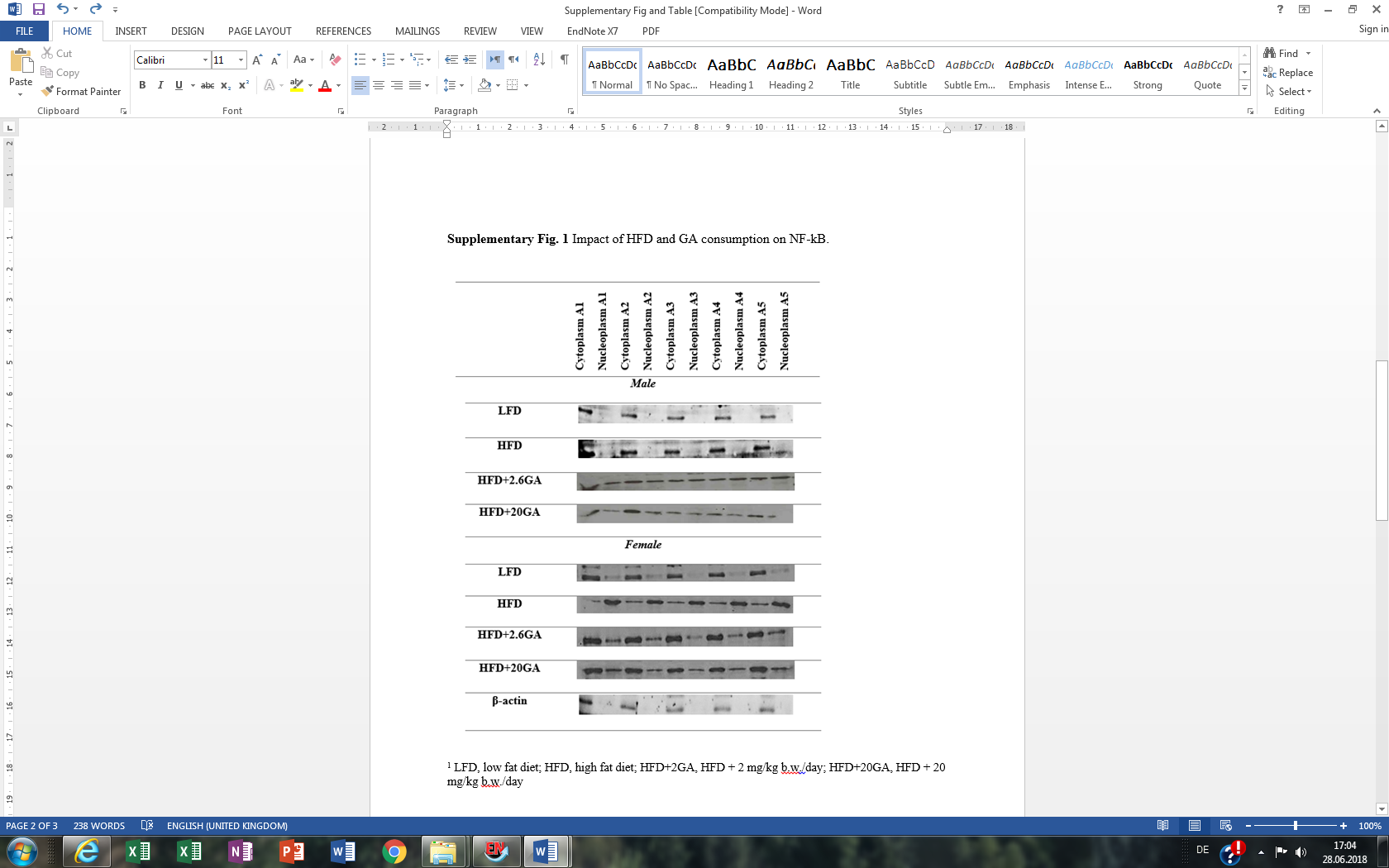


^1^ LFD, low fat diet; HFD, high fat diet; HFD+2GA, HFD + 2 mg/kg b.w./day; HFD+20GA, HFD + 20 mg/kg b.w./day

**Supplementary Table 1.** Composition of the diets**.**

| **Formula** | **High Fat Diet (g/kg)** | **Low Fat Diet**  **(g/kg)** |
| --- | --- | --- |
| Casein | 265.0 | 210.0 |
| L-Cystine | 4.0 | 3.0 |
| Maltodextrin | 160.0 | 100.0 |
| High amylose corn starch | 0.0 | 500.0 |
| Sucrose | 90.0 | 39.14 |
| Lard | 310.0 | 20.0 |
| Soybean oil | 30.0 | 20.0 |
| Cellulose | 65.5 | 35.0 |
| Anhydrous milkfat | 0.0 | 20.0 |
| Mineral mix | 48.0 | 48.0 |
| Calcium phosphate | 3.4 | 0.0 |
| Vitamin mix | 21.0 | 21.0 |
| Choline bitartrate | 3.0 | 2.75 |
| Blue food color | 0.1 | 0.0 |
| Yellow food color | 0.0 | 0.1 |
